# Genes associated with persistent lumbar radicular pain; a systematic review

**DOI:** 10.1186/s12891-016-1356-5

**Published:** 2016-12-13

**Authors:** Siri Bjorland, Aurora Moen, Elina Schistad, Johannes Gjerstad, Cecilie Røe

**Affiliations:** 1 Faculty of Medicine, University of Oslo, 1072 Blindern, Oslo, 0316 Norway; 2 Department of Physical Medicine and Rehabilitation Oslo University Hospital, Ullevål, Postboks 4956 Nydalen, N-0424 Oslo, Norway; 3 National Institute of Occupational Health, Gydas vei 8, Oslo, 0363 Norway; 4 Department of Molecular Bioscience, University of Oslo, 1072 Blindern, Oslo, 0316 Norway

**Keywords:** Lumbar radicular pain, Genes, Biomarkers

## Abstract

**Background:**

The aim of the present study was to provide an overview of the literature addressing the role of genetic factors and biomarkers predicting pain recovery in newly diagnosed lumbar radicular pain (LRP) patients.

**Methods:**

The search was performed in Medline OVID, Embase, PsycInfo and Web of Science (2004 to 2015). Only prospective studies of patients with LRP addressing the role of genetic factors (genetic susceptibility) and pain biomarkers (proteins in serum) were included. Two independent reviewers extracted the data and assessed methodological quality.

**Results:**

The search identified 880 citations of which 15 fulfilled the inclusion criteria. Five genetic variants; i.e., OPRM1 rs1799971 G allele, COMT rs4680 G allele, MMP1 rs1799750 2G allele, IL1α rs1800587 T allele, IL1RN rs2234677 A allele, were associated with reduced recovery of LRP. Three biomarkers; i.e., TNFα, IL6 and IFNα, were associated with persistent LRP.

**Conclusion:**

The present results indicate that several genetic factors and biomarkers may predict slow recovery in LRP. Still, there is a need for replication of the findings. A stricter use of nomenclature is also highly necessary.

**Trial registration:**

The review is registered PROSPERO 20^th^ of November 2015. Registration number is CRD42015029125.

## Background

Low back pain (LBP) has a lifetime prevalence of 70% [1]. The annual prevalence of lumbar radicular pain (LRP) in the population is estimated to 2–3% [2, 3]. Hence, LRP, also referred to as “sciatica”, account for 5–10% of the low back pain conditions. However, the disability is worse and the recovery is slower for LRP than for other low back pain conditions [3, 4]. Low back disorders constitute an important source of disability and are among the most cost-intensive health problems [5].

Development of persistent low back pain and sciatica may be associated with ergonomic strains, but also psychosocial aspects. Risk factors such as age, smoking, body weight, height, occupational load and mental stress contribute to LRP [2, 3, 6–8]. Clearly, many psychosocial factors predict poor recovery in LRP [6, 9]. In addition, genetic variability may influence the risk of a chronic outcome [10, 11].

LRP is characterized by radiating pain that typically follows the dermatome of the affected nerve root from the lumbar or sacral spine [12]. Previous data suggest that discharges emanating from the dorsal nerve roots or their ganglions explain the radiating nature of this form of back disorder [13]. LRP may be induced by mechanical compression of the nerve root, but also by the biochemical influence on the neuronal tissues caused by a local inflammatory process. Moreover, leak of nucleus pulposus from herniated discs may have many effects on the nerves inducing histological changes and increased neuronal excitability. Microvascular changes close to the dorsal ganglion, spinal nerve roots and spinal cord is a part of the pathogenesis [14, 15].

Environmental factors including heavy work load is assumed to contribute to acceleration of degeneration of the spinal joints and discs, but also genetic factors are of importance [10]. It has been postulated that heritability for back pain range from 30 to 45% [16]. The genetic susceptibility for LBP and LRP may be associated with genetic variability in genes related to modulation of nociceptive processing, tissue degeneration and local or systemic inflammation.

In particular genetic variability important for opioid, dopaminergic, adrenergic and serotonergic signaling may affect modulation of nociceptive processing [17–19]. Several previous studies demonstrate a link between genetic variability in the gene encoding opioid receptor mu 1 (OPRM1) and LRP [20, 21]. Earlier reviews, for example Diatchenko et al. [22], highlight that genetic factors related to the enzyme catechol-O-methyltransferase (COMT) affect cortical pain processing and the risk of chronic LBP.

Genetic variability in the gene encoding the sodium ion channel (SCN9A) [23] and the GTP cyclohydrolase 1 (GCH1) [24] gene may affect LRP, indicating that genetic factors may affect peripheral nerves as well. GCH1 is an enzyme involved in production of tetrahydrobiopterin (BH4). BH4 is an essential cofactor for catecholamine, serotonin and nitric oxide production. Earlier data also suggest that disc degeneration and the clinical outcome after sciatica may be associated with the large molecule collagen type IX alpha 2 (COL9A2) [25]. Thus, previous data show possible association between genetic markers and lumbar disc degeneration. However, the relationship between degenerative changes and persistence of pain is still controversial [26, 27].

Interestingly, previous findings [28] suggest that patients with lumbar disc herniation (LDH) have more peripheral Th17 cells and enhanced IL-17 expression in blood compared with healthy controls. Some studies also indicate an association between genetic variability in genes encoding interleukin 1 (IL-1α), interleukin 6 (IL-6) and the human leukocyte antigen II (HLA II) regarding persistent LRP [29–33]. Hence, back pain after disc herniation seems to be associated with activation of the immune system.

From a clinical point of view, slow recovery is a major challenge in LRP – the disability is worse and the recovery is slower for LRP than for LBP. Still, previous reviews have only addressed the relationship between genetic variability and LBP. In the present study, however, we provide an overview of the literature addressing genetic factors and biomarkers predicting pain recovery in LRP patients. The present review emphasizes that several genetic factors and biomarkers described in the literature may predict slow recovery in LRP.

## Methods

### Search strategy

The Medline OVID, Embase, PsycInfo and Web of Science were searched using optimized systematic search strategies including mesh words with explore and a combination of words in the title or abstract related to different expressions of Lumbar radicular pain, Genetic variation and Pain biomarkers. The main key words for the search included “lumbar radicular pain”, “sciatica”, “pain and lumbar disc herniation”, “pain and lumbar prolapse” OR “lumbar radiculopathy”, AND “genetic variability”, “genetic polymorphism”, “allele”, “haplotype”, “microRNA”, “pain biomarker”, “cytokines”, “chemokines”, “interleukins” OR “interferons”. The search was performed from 2004 up to 12^th^ of January 2015.

### Selection of studies

Inclusion criteria were prospective studies, including patients with lumbar radicular pain, and assessing genetic factors or pain biomarkers. Exclusion criteria were non English language, lumbar radicular pain due to tumor, infection or systemic disorders.

### Procedure

Based on screening of the titles and abstracts eligible articles for full text reading by two of the authors were identified.

### Assessing the quality of the studies

A checklist based on Sanderson et al. [34], QATSO (Quality Assessment Tool for Systematic Reviews of Observational studies) [35] and the STROBE statement guidelines (Strengthening the Reporting of Observational Studies in Epidemiology) [36] was used. The checklist compromised seven criteria namely: external validity, sample size, description of sample, follow up rate, appropriate reporting of outcome, adjustment for confounding factors (No = not adjusted for any covariates. Yes = adjusted) and correction for multiple testing. The assessments of the two reviewers were compared. If disagreement a final evaluation of the paper was performed.

## Results

**Fig. 1:**
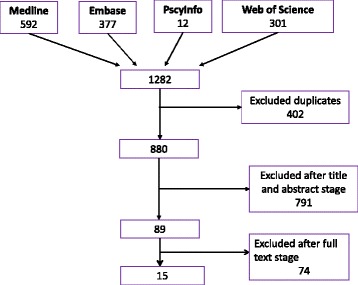
Selection process

The systematic search identified 880 relevant publications, of which 791 were excluded after screening of titles and abstracts. Thus, 89 studies were found eligible, but after full-text screening only 15 publications met the inclusion criteria (Fig. 1).

### Methodological quality

A summary of methodological quality is shown in Table 1. External validity found to be satisfactory in 11 of the 15 included studies and number of cases >100 in 10 of the studies. The studies comprise a total of 872 LRP patients and mean age ranged from 41 to 47 years. Seven of the studies emanates from the same patient population that affect the total number of patients included (Table 1). Although all the studies provided a short description of the sample, several shortcomings in this description were identified. Just one study, Karppinen et al. [29], included BMI and work load in the analyzes. Moreover, Gebhardt et al. [37] controlled for smoking and BMI. In only 6 studies the data were evaluated after correction for their multiple testing.

**Table 1:** Methodological quality assessment of included studies

Study	External validity	Sources of Bias				Statistics	
	Findings can be generalised	Sample size cases	Description sample	Follow up rate	Appropriate outcomes reported	Adjustment confounding	Correction multiple testing
Andrade et al. (2013) [39]	No	*n* = 10	Yes	100%	Yes	No	No
Andrade et al. (2011) [38]	No	*n* = 10	Yes	100%	Yes	No	No
Gebhardt et al. (2006) [37]	Yes	*n* = 31	Yes	88%	Yes	Yes	Yes
Hasvik et al. (2014) [21]	Yes	*n* = 118^a^	Yes	95%	No	Yes	Yes
Jacobsen et al. (2013) [44]	Yes	*n* = 260^a^	Yes	91%	Yes	Yes	Yes
Jacobsen et al. (2012) [43]	Yes	*n* = 258^a^	Yes	89%	Yes	Yes	Yes
Karppinen et al. (2008) [29]	Yes	*n* = 153	Yes	97%	Yes	No	No
Moen et al. (2014) [31]	Yes	*n* = 252^a^	Yes	91%	No	Yes	Yes
Olsen et al. (2012) [20]	Yes	*n* = 258^a^	Yes	92%	Yes	Yes	Yes
Rut et al. (2014) [40]	Yes	*n* = 176	Yes	100%	Yes	Yes	No
Schistad et al. (2014) [42]	Yes	*n* = 108^a^	Yes	90%	Yes	Yes	No
Schistad et al. (2014) [30]	Yes	*n* = 121^a^	Yes	91%	No	No	No
Scuderi et al. (2009) [45]	No	*n* = 47	Yes	100%	Yes	No	No
Takeuchi et al. (2007) [41]	No	*n* = 27	Yes	100%	Yes	Yes	No
Tegeder et al. (2006) [24]	Yes	*n* = 168	Yes	88%	Yes	Yes	NA

*NA*, not applicable, *ND*, not described

^a^emanates from the same patient population

### Assessment and definition of pain recovery

All but one study reported VAS (Visual analog scale) as assessment tool for pain. Tegeder et al. [24] did not describe how pain was measured, but provide us the z-score from several time points to express the development in pain intensity over time. Detailed information about pain intensity was not present in most of the included studies. Moreover, we found a variety of different pain descriptions/locations and procedures for pain testing. In two studies, the pain score was based on pain during activity, in two of the studies at rest, whereas in the rest of the studies this was not clearly described. Pain duration at baseline were described in five studies where two reported duration ≥3 months, one study <3 months and the two last both ≥3 and <3 months.

Even if the follow up time was 12 months or more in 12 of the studies, the presentation of the development of pain over time was not clear. Gebhardt et al. [37] measured pain at 12 time points and gave a detailed description of how high-sensitive C-reactive protein (hsCRP) declined corresponding to decreased pain first 3 weeks, but did not emphasized what happened after the subacute phase.

Pain recovery was described in 4 of the studies. Andrade et al. [38, 39] used >20% reduction in VAS while Rut et al. [40] and Takeuchi et al. [41] used >50% reduction in VAS between baseline and follow to define recovery. Specific description of change in pain state during the follow up period was reported in just two of the studies. Both Olsen et al. [20] and Schistad et al. [30, 42] described a significant decrease in pain the first year after herniation.

### Genetic variability and pain recovery

In 9 of the studies the association between genetic variability and LRP were studied (Table 2). The roles of 20 genetic polymorphisms were addressed. Only 1 study addressed the relationship between genetic variability and tissue degeneration seen on MRI.

**Table 2:** Genetic variability and pain recovery

Gene	rs number	Base substitution	Position in DNA	Amino acid substitution	Reference	Result
OPRM1	rs1799971	A→G	118	Asn40Asp	Hasvik et al. (2014) [21]	↑(W) ↓(M)
	rs1799971	A→ G	118	Asn40Asp	Olsen et al. (2012) [20]	↑(W) ↓(M)
COMT	rs4680	A→ G	472	Val158Met	Jacobsen (2012) [43]	↑
	rs4680	A→ G	158	Val 158Met	Rut et al. (2014) [40]	↓
	rs6269	G→ A	−98	–		–
	rs4633	T→ C	186	–		↓
	rs4818	G→ C	408	–		–
MMP1	rs 1799750	1G allele→ 2G allele	−1719	–	Jacobsen (2013) [44]	↑
IL1A	rs1800587	C→ T	−949	–	Moen et al. (2014) [31]	↑
	rs1800587	C→ T	−949	–	Schistad et al. (2014) [30]	↑
IL1B	rs1143627	T→ C	−118	–	Moen et al. (2014) [31]	–
IL1RN	rs2234677	G→ A	−87	–		↑
IL-6	rs1800797 rs1800796	A→ G	−661	–	Karppinen et al. (2008) [29]	- (Haplotype GGGA)
		G→ C	−636	–		
	rs1800795	G→ C	−237	–		
	rs13306435	T→ A	486	Asp162Glu		
GCH1	rs8007267	G→ A	−9610	–	Tegeder et al. (2006) [24]	↓ (Haplotype ATCA)
	rs3783641	A→ T	343 + 8900	–		
	rs8007201	T→ C		–		
	rs752688	G→ A	509 + 1551 627–708	–		

The rs number refers to a specific SNP and rs stands for Reference SNP cluster ID, created by National Center for Biotechnology Information (NCBI) (Ref.: http://www.ncbi.nlm.nih.gov/). Base substitution refers to replacement of one base with another in DNA. Position on DNA based on information from NCBI. Only two of our SNP causes a direct amino acid substitution. A change of nucleotide in the exon is a prerequisite for change in amino acid. A replacement of nucleotide in the intron does not cause such a substitution but may have a role in the transcription process. Polymorphism located in the promotor part of the gene is expressed by adding minus prior to the position on the DNA

↑ (positive association with poor recovery), ↓ (negative association with poor recovery), - (no change in amino acid), - (no association with poor recovery), *W* (Women), *M* (Men)

Olsen et al. [20] and Hasvik et al. [21] demonstrated that a genetic variant, OPRMI rs1799971 SNP, in the gene encoding OPRM1 receptor is associated with both pain and subjective health in LRP patients. The OPRM1 rs1799971 G allele increased the pain score in women, but reduced the pain score in men. Thus, the data revealed a significant interaction between sex and OPRM1 genotype regarding the pain intensity.

Jacobsen et al. [43] showed that the COMT rs4680 SNP affects pain recovery after disk herniation. In both men and women, carriers of COMT rs4680 2G alleles had more pain than carriers of two A alleles at 6 months after disc herniation. Conversely, Rut et al. [40] reported that carriers of two COMT rs4680 G alleles may be associated with significant positive improvement in pain recovery one year after surgery.

Jacobsen et al. [44] addressed the relationship between MMP1 rs1799750 SNP and tissue degeneration. The data indicated that the MMP1 rs1799750, in the gene encoding the MMP1 enzyme, may affect the long-term outcome in disc herniation patients. Carriers of two MMP1 rs1799750 2G alleles had a reduced pain recovery rate, but not increased MRI disc changes.

Moen et al. [31] and Schistad et al. [30, 42] found increased risk of persistent pain in carriers of the IL1α rs1800587 T allele. Moreover, Karppinen et al. [29] demonstrated a significant association between the IL-6 haplotype rs1800797 G/rs1800796 C/rs1800795 C/rs13306435 A and days of leg pain 3 years after disc herniation in men with high physical work load. Finally, Tegeder et al. [24] showed that the GTP cyclohydorlase (GSCH1) haplotype rs8007267 A/rs3783641 T/rs8007201 C/rs752688 A could be protective and be associated with less pain following discectomy.

Six of the studies emanates from the same patient population (Table 1). None of these association studies included data on protein expression.

### Biomarkers and pain recovery

Six studies presented data on biomarkers linked to pain recovery (Table 3). As many as 28 biomarkers have been assessed: IL1b, IL-2, IL-4, IL-5, IL-6, IL-7, IL-8, IL- 10, IL-12, IL-13, IL-17, G-CSF, GM-CSF, MCP-1b, MIP-1b, TNFα, TNF R1, TNF R2, CGRP1, Galanin, Neuropeptides4, SubstP. Most of the biomarkers examined are members of the cytokine family, but also the role of some neuropeptides is among the studied molecules. In addition low levels of the C-reactive protein (hsCRP) were assessed in the study by Gebardt et al. 2006 [37].

**Table 3:** Characteristics of the included studies

Study	Cases	Pain local.	Pain time points	Gene	Biomarker	Results
Andrade et al. (2013) [39]	*n* = 10	Leg	1 day preoperative 6 weeks postoperative 12 month postoperative		(Tissue: PM, AF, NP) IL-1beta, IL-6	– –
Andrade et al. (2011) [38]	*n* = 10	Leg	1 day preoperative 6 week postopertive 12 mont postoperative		(Tissue: PM, AF, NP) TNF alfa TNF R1 TNF R2	↑ (6 week *r* = 0.54, 12 month *r* = 0.65) ↑ (6 week *r* = 0.75, 12 month *r* = 0.80) ↓ (6 week *r* = -0.60, 12 month *r* = -0.60)
Gebhardt et al. (2006) [37]	*n* = 31	ND	0 day, 3 day, 7 day, 10 day, 14 day, 17 day, 21 day, 2,3 month, 6 month		(Blood) hsCRP	↓ (first 3 weeks *p* < 0.05) - (after 3 weeks)
Hasvik et al. (2014) [21]	*n* = 118	Leg	Baseline 12 month	OPRM1		↑ (Women *p* < 0.008) ↓ (Men *p* < 0.008)
Jacobsen et al. (2013) [44]	*n* = 260	Leg Back	Baseline 6 week 12 month	MMP1		↑ (6 week *p* = 0.004, 12 month *p* = 0.004)
Jacobsen et al. (2012) [43]	*n* = 258	ND Activity	Baseline 6 month postoperative 12 month postoperative	COMT		↑ (6 month *p* = 0.028)
Karppinen et al. (2008) [29]	*n* = 153	Leg Back	Baseline 6 week 6 month 12 month	IL-6		–
Moen et al. (2014) [31]	*n* = 252	Activity	Baseline 6 week 6 month 12 month	1 L-1a + IL-1RN IL-1b + IL-1RN		↑ (12 month *p* = 0.049) –
Olsen et al. (2012) [20]	*n* = 258	Leg Back Activity	Baseline 6 week 6 month 12 month	OPRM1		↑ (12 month Women *p* = 0.002) ↓ (12 month Men *p* = 0.002)
Rut et al. (2014) [40]	*n* = 176	Leg Back	Preoperative 12 month postoperative	COMT		↑ (12 month *p* = 0.0042)
Schistad et al. (2014) [42]	*n* = 108	Leg Back Activity Rest	Baseline 12 month		(Blood) IL-6	↑ (12 month *p* = 0.004)
Schistad et al. (2014) [30]	*n* = 121	Leg Back Activity Rest Present	Baseline 6 week 12 month	IL-1a		↑ (12 month *p* = 0.002)
Scuderi et al. (2009) [45]	*n* = 47	ND	Preinjection 3 month postinjection		(CSF) IFNa IL2,4,5,6,7,8,10,12,13,17 G-CSF GM-CSF TNFa IL1b MCP-1b MIP-1b	↓ (3 month *p* = 0.001) – – – – – – –
Takeuchi et al. (2007) [41]	*n* = 27	Leg	Preoperative 3 week postoperative		(Blood) CGRP1 Galanin Neuro-peptide4 SubstP	↑ (Preoperative *p* = 0.01) – – –
Tegeder et al. (2006) [24]	*n* = 168	ND	4 time points^a^	GCH		↓ (*p* < 0,05)

↑ (positive association with poor recovery), ↓ (negative association with poor recovery), - (no association with poor recovery), *ND* (not defined, ^a^(z-score). All pain measures reported by VAS except Tegeder et al. *PM* (Paravertebral muscle), *AF* (Annulus fibrosis), *NP* (Nucleus pulposus)

Andrade et al. [39] was unable to detect any link between Il-6 and pain recovery while Schistad et al. [30, 42] demonstrated from the results that high level of IL-6 correlate with less favorable pain recovery 1 year after disk herniation. Regarding recovery, Andrade et al. [38] and Scuderi et al. [45] found a link to tissue and CSF level of TNFα at one year and tissue IFNα level at 3 month – whereas Takeuchi et al. [41] found that plasma level of the neuropeptide CGRP was associated with the extent of sciatica. In acute lumbosciatic patient, hsCRP declined with decreased pain the first 3 weeks after disc herniation, but no clear relationship between pain and level of hsCRP was observed after that (Gebhardt et al. 2006 [37]). Specific results from the studies assessing biomarkers and pain recovery are listed in Table 3.

## Discussion

In the present review, we identified nine studies addressing the relationship between genetic polymorphism and LRP. The data analyzed in these studies were limited to eleven DNA base substitutions. In all these studies, polymorphisms of genes encoding proteins expected to affect the phenotype were studied [46]. Some of the SNPs were located in the promoter region, whereas others were located in the coding regions of the genes.

Two studies reported a positive association between the OPRM1 SNP rs179971 and poor recovery of pain in women with LRP [20, 21]. These data support the previous observation that some individuals, in particular in females, carrying the ORPM1 G allele have increased pain sensitivity [47, 48]. OPRM1 is crucial for processing and modulation of pain. Moreover, several studies addressed the association between COMT SNP rs4680 G allele and pain. This enzyme metabolizes catecholamines and thus modulates adrenergic, noradrenergic and dopaminergic signaling in the CNS as well as in the peripheral tissue. However, while Jacobsen et al. found a positive correlation between the COMT rs4680 G allele and long lasting pain, Rut et al. reported that the same SNP may be associated with better clinical outcome [40, 43].

Although the data may be debated, most experimental studies support a positive correlation between the COMT haplotype rs4680 G, rs6269 A, rs4633 C, rs4818 C and pain hypersensitivity [49]. Moreover, several of these COMT SNPs may be associated with increased postoperative pain. For example, the COMT haplotype rs4680 G, rs6269 A, rs4633 C, rs4818 C is associated with slower recovery after surgical treatment for lumbar degenerative disc disease [50].

Only one study addressed the relationship between genetic variability, tissue degeneration and persistent pain [44]. Previous data suggest that the enzyme MMP influences tissue degradation or inflammation [51]. Surprisingly, however, no relationship between the MMP1 SNP rs1799750 and disc degeneration shown on MRI was observed in the systematic search performed for this review. Still, the study of Jacobsen et al. [44] showed that the MMP1 SNP rs1799750, i.e., the 2G allele insert, may be associated with poor pain recovery after lumbar disc herniation. Previous studies show that other painful degenerative inflammatory conditions may be associated with the MMP1 SNP rs1799750 2G allele [52, 53].

Several lines of evidence suggest that genetic variability in genes encoding inflammatory cytokines may be associated with persistent LBP [16]. The present review shows data that the IL1α rs1800587 T allele and the IL6 haplotype rs180077 G, rs1800796 C, rs1800795 C, rs13306435 A may be associated with slower recovery in LRP patients [29–31]. Moreover, data exists that the rare allele of the gene encoding the GTP cyclohydrolase, could be associated with reduced pain following discectomy in LRP patients [24]. However, more recent reports questions these data [54].

Six studies in the present review show correlations between protein levels and recovery of pain [37–39, 41, 42, 45]. However, only IL-6, TNFα and IFNα seem to be associated with persistence of LRP. Schistad et al. [30, 42] showed that higher serum level of IL-6 predicts a less favorable clinical outcome. Moreover, Scuderi et al. [45] and Andrade et al. [38] showed that TNFα and IFNα may be associated with persistent LRP. In addition, previous studies suggest a correlation between TNFα and recovery of pain in chronic LBP and lumbar radiculopathy patients [55, 56].

Development of persistent pain is multifactorial. It is now well established that psychosocial factors, such as depressive mood, distress and somatization, may contribute to chronic LRP [57]. Together with individual factors as gender, age, smoke, obesity and education level, genetic predisposition may be crucial prognostic factors in LBP patients as well as LRP patients [6, 57].

### Strength and limitation

To our knowledge, this is the first paper attempting to provide an overview over genetic variants linked to the development of persistence LRP. Still, many of the findings, including the role of the GTP cyclohydrolase, are controversial and need to be replicated [54]. In addition few researchers present genetic data together with changes in protein expression. In further studies this knowledge gap need to be highlighted.

Therefore, the interpretations of the data, but also the heterogeneity in the nomenclature, might be challenging. In the present review we have listed the genetic variants by number, base substitution, position on DNA and if applicable amino acid substitution. Position of base replacement refers to position found in National Center for Biotechnology (NCBI). The majority of the nucleotide replacements listed is located in the intron or promotor region. Only two of the SNPs cause amino acid substitution. Regarding the interpretation of the data, the link between the genetic variability, protein expression and function is therefore definitely challenging.

External validity in all but one of the genetic association studies is fair. The sample size was >100 patients in nine studies, however, as many as six of the samples emanate from the same cohort, and the methodological quality of the studies may still be debated. A bias towards only positive findings being published cannot be excluded. Moreover, the external validity is poor in the six studies about biomarkers – and in only two studies correction for multiple testing was performed. The strength of this review, however, is the optimized systematic search in several databases and the strict inclusion criteria.

Unfortunately, the studies were too few and too heterogeneous to perform meta-analyses, and many of the studies emanate from the same cohort. Further on, GWAS would shed light on other genetic factors related to the same phenomena. Unfortunately, however, most clinical studies do not have enough statistical strength for GWAS. This may be a major challenge in clinical research. None of the included studies were GWAS. Moreover, no studies addressed the interaction between environmental factors and genetic markers. An extensive systematic review by Eskola et al. 2012 regarding LBP and genetics evaluated that the credibility of reported genetic associations were mostly weak including four of our candidate genes; IL1α, IL1β, IL1RN, MMP1. Finally, each SNP in this review explained just about 1% of the variance. Previous studies show that the explained variance of the SNPs in general is rather low – even for inherited characteristics like human height [58]. Thus, the low explained variance in the present studies underscores the complex mechanisms and multifactorial nature of LRP. Furthermore, the causal relationship between genetic factors and LRP remains to be examined. The clinical value of this review can be questioned but the presented findings may be of importance for better understanding pain mechanism and further research.

## Conclusion

This systematic review suggests that several genetic factors involved in pain perception, inflammation and tissue degeneration may be linked to poor recovery in LRP patients. Further, serum levels of the IL-6, IFNα and TNFα proteins correlate with persistent LRP. The existing literature in this review revealed, however, that many articles are based on the same cohorts; hence the results were generally not replicated in different cohorts. Relatively few candidate genes were examined and the explained variance relatively low. Hence, broader panels of genes and replication of findings across pain cohorts are needed in order to implement these findings in diagnostic procedures and treatment.

## Abbreviations

COMT: Catechol-O-methyltransferase
hsCRP: High-sensitive C-reactive protein
IFN: Interferon
IL: Interleukin
LBP: Low back pain
LRP: Lumbar radicular pain
MMP: Matrix metalloproteinase
OPRM1: Opioid receptor mu 1
SNP: Single nucleotide polymorphism
TNF: Tumor necrosis factor
VAS: Visual analogue scale
